# Lacking a vocational upper-secondary diploma: motivational and proximal contextual predictors in primary and lower-secondary education

**DOI:** 10.1186/s40461-025-00177-5

**Published:** 2025-01-18

**Authors:** Jan Hofmann, Markus P. Neuenschwander, Lukas Ramseier

**Affiliations:** Center for Learning and Socialization, University of Applied Sciences and Arts Northwestern Switzerland, Bahnhofstrasse 6, Windisch, 5210 Switzerland

## Lacking an upper-secondary diploma

In Switzerland, the confederation, cantons, and professional organizations have set the education policy objective that 95% of adolescents should have an upper-secondary certificate or diploma^1^ by the age of 25 (i.e., about 10 years after completing compulsory school, at the transition from adolescence to adulthood; Swiss Coordination Centre for Research in Education 2023; United Nations 2011). Other countries have similar upper-secondary completion rate policies (Reiling and Strøm 2015). Individuals who are without an upper-secondary diploma in adulthood are increasingly becoming a risk group for unemployment, social welfare dependency, and undersupplying the labor market with qualified individuals (Häfeli and Schellenberg 2009). Research indicates that those risks surface not just on the verge of adulthood but also earlier (Meyer 2018). From the fifth year after completion of compulsory school, stability effects start to emerge regarding adolescents’ educational situation. Meyer (2018) found that adolescents who lack an upper-secondary diploma 5 years after completing compulsory school (e.g., who are employed but work without any upper-secondary diploma) are most likely in the same situation 1 year later and in the years thereafter. Their probability of graduating at the upper-secondary level dwindles year after year because those who do not obtain an upper-secondary diploma in adolescence or young adulthood are unlikely to do so later in their careers (Meyer 2018).

In general, adolescents try to avoid falling into this risk group, and achieving an upper-secondary diploma is one of their top educational goals (Ackermann and Benz 2023). It is therefore vital to analyze predictors of lacking an upper-secondary diploma, as we did for the situation 5 years after completion of compulsory education in Switzerland.

## Upper-secondary education in the swiss education system

Together with compulsory education, upper-secondary education forms a central part of the Swiss education system. Upper-secondary education follows compulsory school, which is mandatory for all children and is divided into primary education and lower-secondary education. In most cantons, primary education starts when students are about 6 years of age (Grade 1) and takes 6 years, ending in Grade 6. Lower-secondary education divides students into school tracks based on their achievements and usually takes 3 years, ending in Grade 9. On average, students transition to upper-secondary education at age 15 or 16. Upper-secondary education can be roughly broken down into academic/general education and vocational training. Both paths lead to a qualifying upper-secondary diploma, which is typically received after 2 to 4 years of training. While academic/general education imparts knowledge to prepare adolescents for tertiary level education programs, vocational training offers adolescents the opportunity to acquire skills and knowledge needed to work in a given occupation (Swiss Conference of Cantonal Ministers of Education 2024). Therefore, the Swiss upper-secondary education system is similar to other European education systems which also have the two tracks, academic/general education and vocational training. Academic/general education takes place in either baccalaureate schools or upper-secondary specialized schools. Vocational education takes the form of vocational education and training (VET). Adolescents either learn full-time in a vocational school, or they are in a dual VET program with two learning contexts, training company and vocational school. In both VET settings, adolescents have to sign an apprenticeship contract. Whereas adolescents in fully school-based vocational training sign a contract with their vocational school, dual-VET apprentices sign a contract with their training company (Zulauf et al., 2008). The contract is fix-termed for the duration of the vocational education program, but either of the contractual partners can request a premature contract termination provided that all mediation attempts by the cantonal authorities proved unsuccessful (Swiss Service Center for Vocational Education and Training 2022). Like adolescents in academic/general upper-secondary education who have to pass their final examinations to complete training, adolescents in vocational training are required to pass the qualification procedure to complete training. In a final examination, apprentices’ competences and skills are tested. Multiple attempts in the qualification procedure are possible, but apprentices may not repeat their final examination more than twice in the same training program (Swiss Service Center for Vocational Education and Training 2022). Adolescents in academic/general education and those in vocational education acquire their upper-secondary diploma between the ages of 18 and 22 provided they follow straight educational pathways.

## Lacking a vocational upper-secondary diploma

More than two thirds of compulsory school completers transition to vocational upper-secondary education in Switzerland (Swiss Coordination Centre for Research in Education 2023). Therefore, lacking an upper-secondary diploma, for most adolescents, means lacking a vocational upper-secondary diploma. In this field of research, we identified two research gaps. First, we could not find any studies that investigated the extent to which two of the main events indicating nonlinear career trajectories at upper-secondary level—premature contract termination and failed attempts in the qualification procedure—predict the probability of lacking an upper-secondary diploma. To develop targeted educational policy measures to reduce noncompletion rates, it is crucial to know which of these two predictors explains more variance. Second, VET-dropout research lacks long-term studies about the predictors of lacking an upper-secondary diploma (Krötz 2024). There is a research gap concerning motivational and proximal contextual (e.g., family/parents) factors in primary and lower-secondary education. Compared to distal contextual factors, motivational and proximal contextual factors provide intervention possibilities that are easier to implement. At the primary and lower-secondary levels, interventions can be initiated at an early career stage. We targeted both research gaps with two research questions:

1) To what extent can the lack of a vocational upper-secondary diploma 5 years after completion of compulsory school be predicted by two of the main events indicating nonlinear career trajectories at upper-secondary level—premature contract termination and failed attempts in the qualification procedure?

2) To what extent can motivational and proximal contextual factors in primary education and motivational factors in lower-secondary education predict the lack of a vocational upper-secondary diploma 5 years after completion of compulsory school?

Answering these research questions will help clarify the interrelationships among premature contract terminations, failed attempts in the qualification procedure, and lacking a vocational upper-secondary diploma. Investigating motivational and proximal contextual factors in primary and lower-secondary education allows for early intervention possibilities to reduce the rate of individuals who struggle to complete upper-secondary education.

## Research on lacking a vocational upper-secondary diploma

Studies on adolescents’ situation of lacking a vocational upper-secondary diploma some years after completing compulsory school are scarce (e.g., Stalder et al. 2008). However, under the umbrella term “dropout,” a plethora of studies have dealt with similar problems (e.g., Blondal and Adalbjarnardottir 2009, 2014; Böhn and Deutscher 2022; Findeisen et al. 2023; Holtmann and Solga 2023; Krötz 2024; Krötz and Deutscher 2022; Michaelis and Richter 2022; Schmid 2017; Vasalampi et al. 2010). Although studies are manifold, there is no clear-cut definition of the term “dropout” due to its complex and multifactorial nature (Krötz 2024). Definition ambiguities arise, particularly in the field of VET research. Many VET researchers have used the term “dropout” synonymously with “premature contract termination” (e.g., Böhn and Deutscher 2022) and defined “dropping out” as an act by apprentices “who leave training prematurely without achieving a formal qualification, regardless of the causes, the initiator, and whether a training contract with the training company needed to be terminated” (Krötz 2024, p. 4). For this kind of dropout, a meta-synthesis indicated low training wage, mismatch between desired and actual apprenticeship occupation, low educational level, poor performance in training, learning disability, increasing age, and migration background as main predictors of dropping out (Böhn and Deutscher 2022). However, this definition does not include the whole dropout process. According to Krötz (2024), the dropout process occurs in three phases: development, decision, and adjustment. Whereas the aforementioned definition covers the first two phases, the adjustment phase is ignored. We argue that a definition of “dropout” from VET should also incorporate individuals’ actions after they prematurely leave vocational training (adjustment) because the term implies a somewhat permanent withdrawal from VET and, in a wider sense, from formal qualification. The literature includes terms such as “actual dropout” and “permanent dropout” to refer to this phenomenon (Findeisen et al. 2023; Holtmann and Solga 2023). However, because it is possible to return to qualifying upper-secondary education at any time (which turns individuals from “dropouts” to “stopouts”; Holtmann and Solga 2023), the word “permanent” carries some problems, too. Therefore, researchers often used the term “downward dropout” (Krötz and Deutscher 2022), which occurs when individuals become unemployed or work in jobs without formal qualification after prematurely leaving vocational training (Krötz and Deutscher 2022). According to Holtmann and Solga (2023), individuals who dropped out for at least 1 training year had low math competencies and a poor match between their VET program and their desired occupation. Krötz and Deutscher (2022) reported that training quality and adolescents’ educational level were the main drivers for high downward dropout intentions. Regarding this study’s aim, the definition of “downward dropout” is also only partially satisfactory because someone can be in the situation of not having any upper-secondary diploma years after completing compulsory school without being a downward dropout (e.g., when someone has changed training occupations or training companies multiple times but still is in a VET program 5 years after completing compulsory school). Therefore, the present study also contributes to this rather underexplored field in dropout research.

## Lacking a vocational upper-secondary diploma and the social–cognitive career theory of work satisfaction and well-being

To answer both research questions, the social–cognitive career theory of work satisfaction and well-being (SCCT; Lent and Brown 2008) provided the theoretical foundation. Originally developed to predict work satisfaction and overall life satisfaction, the theory has formed the theoretical foundation of various studies and offers an approach to investigate our research questions (Brown and Lent 2019). The theory postulates that goal progress is the main predictor of work satisfaction. Goal progress, in turn, depends on self-efficacy expectations and goal- and efficacy-relevant environmental supports, resources, and obstacles. In the present study, we specifically utilized these theoretical postulations about goal progress and its precursors.

### Goal progress and lack thereof in VET

In VET contexts, adolescents usually pursue the goal of achieving at least an upper-secondary diploma (Ackermann and Benz 2023). Prematurely terminating contracts, having failed attempts in the qualification procedure, and ultimately lacking an upper-secondary diploma all indicate a lack of goal progress at the upper-secondary level. Yet, there is a distinct temporal distance between indicators: Prematurely terminating contracts and having failed attempts in the qualification procedure both precede lacking an upper-secondary diploma. With every premature contract termination, the probability of lacking an upper-secondary diploma increases. The same applies for having one or more failed attempts in the qualification procedure. Whereas about one in four apprentices may experience the termination of an apprenticeship contract in the course of education (Swiss Federal Statistical Office [SFSO], SFSO 2023a), failed attempts in the qualification procedure are less common (about 9%; SFSO 2023b). Multiple occurrences per person are also much more common for premature contract terminations than for failed attempts in the qualification procedure. Over 4 years, 4% of a cohort had two or more premature contract terminations (SFSO 2023a). In comparison, < 1% of this study’s sample had more than one failed attempt in the qualification procedure. Therefore, of the two, premature contract terminations represent the bigger threat for not graduating at the upper-secondary level in the long run (Schmid 2017). To answer our first research question, we tested the following assumptions:

#### Hypothesis 1:

The more premature contract terminations adolescents have (i.e., the higher the number of premature contract terminations), the greater is the probability that they lack an upper-secondary diploma 5 years after completing compulsory school (H1a).

Having one or more failed attempt(s) in the qualification procedure increases the probability that adolescents lack an upper-secondary diploma 5 years after completing compulsory school (H1b).

Premature contract terminations’ effect is significantly bigger than the effect of failed attempt(s) in the qualification procedure (H1c).

### Predictors of goal progress and lack thereof in VET

#### Educational effort

According to Lent and Brown (2008), (lacking) goal progress partly depends on self-efficacy expectations. *Self-efficacy expectations* are personal beliefs about one’s ability. High self-efficacy promotes and sustains efforts to achieve one’s work goals. Effortful individuals are willing to engage in challenging situations and typically spend more time trying to achieve their goals (Stangl 2024). Regarding the educational setting of VET, progress in educational goals, such as earning an upper-secondary diploma, is expected to depend mainly on high levels of educational effort. Vice versa, having low educational efforts (i.e., low willingness to engage in challenging educational situations) should result in a lack of goal progress. Regarding our second research question, SCCT suggests that educational effort is a relevant motivational factor that as early as lower-secondary education (i.e., before VET entry) could affect the lack of goal progress in upper-secondary education, as indicated by premature contract terminations, failed attempts in the qualification procedure, and ultimately lack of an upper-secondary diploma 5 years after completion of compulsory school. We argue that educational effort in lower-secondary education takes the role of a precursor of educational effort in upper-secondary education due to high levels of relative stability in educational effort over time (Neuenschwander et al. 2024b; Vasalampi et al. 2010). For apprentices in Switzerland and students in Finland, respectively, Findeisen et al. (2023) and Vasalampi et al. (2010) found that (goal) effort in lower-secondary school predicts (a lack of) goal progress in upper-secondary education. To answer our second research question, we tested the following assumptions:

##### Hypothesis 2

The greater adolescents’ educational effort in lower-secondary education is, the lower is their number of premature contract terminations (H2a) and the lower is their probability of having one or more failed attempt(s) in the qualification procedure (H2b).

Adolescents’ educational effort in lower-secondary education has a negative indirect effect on the probability of lacking an upper-secondary diploma 5 years after completion of compulsory school, simply mediated by the number of premature contract terminations (H2c) and by having one or more failed attempt(s) in the qualification procedure (H2d).

#### Self-efficacy expectations

Because effort predicts the lack of goal progress and self-efficacy expectations predict effort, the SCCT postulates that effort mediates the effect of self-efficacy expectations on lack of goal progress (Lent and Brown 2008). Regarding our second research question, SCCT suggests that self-efficacy expectations are a relevant motivational factor for educational effort and indirectly lead to lack of goal progress in upper-secondary education (premature contract terminations, failed attempts in the qualification procedure, lack of an upper-secondary diploma). Neuenschwander et al. (2024b) found positive associations between general self-efficacy and educational effort in lower-secondary education. Similarly, Vasalampi et al. (2010) found that self-esteem (a similar concept to general self-efficacy) positively correlates with (educational) goal effort among lower-secondary education students. Based on these findings and regarding our second research question, we assumed that general self-efficacy in primary education (i.e., general beliefs in one’s ability to respond to and control environmental demands and challenges; Schwarzer and Jerusalem 1995) indirectly affects lack of goal progress in upper-secondary education via effort in lower-secondary education. We argue that general self-efficacy’s effect in primary education on educational effort in lower-secondary education partially represents general self-efficacy’s effect in lower-secondary education on educational effort in lower-secondary education due to high levels of relative stability in general self-efficacy over time (West et al. 2020). We tested the following hypotheses:

##### Hypothesis 3

The greater primary school children’s general self-efficacy is, the greater is their educational effort as adolescents in lower-secondary education (H3a).

Primary school children’s general self-efficacy has a negative indirect effect on the probability of lacking an upper-secondary diploma 5 years after completion of compulsory school, serially mediated by educational effort in lower-secondary education and the number of premature contract terminations (H3b) and serially mediated by educational effort in lower-secondary education and having one or more failed attempt(s) in the qualification procedure (H3c).

#### Education-attainment value

According to SCCT, “perceiving that one is efficacious at valued tasks*”* (Lent and Brown 2008, p. 15) is relevant when looking at the predictors of effort and (lack of) goal progress. Individuals show effortful behaviors in situations to which they previously have assigned a high value. For the educational setting of VET, it is the educational value that predicts educational effort and the lack of goal progress in upper-secondary education. According to Eccles and Wigfield (2002), the education-attainment value is a key value in educational contexts. Education-attainment value describes the importance individuals ascribe to formal education. Regarding our second research question, SCCT suggests that education-attainment value is a relevant motivational factor for educational effort and therefore indirectly leads to lack of goal progress in upper-secondary education (premature contract terminations, failed attempts in the qualification procedure, lack of an upper-secondary diploma). In a longitudinal study, Tuominen et al. (2020) reported positive correlations between mastery-extrinsic goal orientation in primary school (a similar concept to the education-attainment value) and school engagement in lower-secondary school (a similar concept to educational effort) among Finnish students. Therefore, like general self-efficacy, education-attainment value may already in primary education indirectly affect upper-secondary (lack of) goal progress via lower-secondary educational effort. We reason that education-attainment value’s effect in primary education on educational effort in lower-secondary education partially represents education-attainment value’s effect in lower-secondary education on educational effort in lower-secondary education due to high levels of relative stability in education-attainment value over time (Tuominen et al. 2020). We tested the following assumptions:

##### Hypothesis 4

The higher primary school children’s education-attainment value is, the bigger is their educational effort as adolescents in lower-secondary education (H4a).

Primary school children’s education-attainment value has a negative indirect effect on the probability of lacking an upper-secondary diploma 5 years after completion of compulsory school, serially mediated by educational effort in lower-secondary education and the number of premature contract terminations (H4b) and serially mediated by educational effort in lower-secondary education and having one or more failed attempt(s) in the qualification procedure (H4c).

#### Parental responsiveness

According to SCCT, proximal contextual factors, such as goal- and efficacy-relevant environmental supports, resources, and obstacles aide or hinder goal pursuit and progress. Additionally, supportive environmental influences help inform self-efficacy through modeling, encouragement, or performance feedback, and self-efficacy, in turn, affects goal progress via effort (Lent and Brown 2008). Research on environmental support’s effects on self-efficacy, effort, and goal progress has shown that parents are one of the main sources of environmental support when it comes to occupational and school matters among early adolescents (Jodl et al. 2001). Parental responsiveness is considered a key supportive factor (Wild 2001). It is a parenting style dimension and defined as “the extent to which parents intentionally foster individuality, self-regulation, and self-assertion by being attuned, supportive, and acquiescent to children’s special needs and demands” (Baumrind 1991, p. 62). In a cross-sectional study, Wild (2001) found positive correlations between parental responsiveness and students’ intrinsic motivation in lower-secondary education. For the context of fully school-based (vocational) upper-secondary education, longitudinal data showed that authoritative parenting practices (which are characterized by high levels of parental responsiveness) negatively predict compulsory-school children’s school disengagement and their lack of goal progress in upper-secondary education, such as the risk of school dropout (Blondal and Adalbjarnardottir 2009, 2014). Based on these findings and theoretical assumptions in SCCT, we assumed an influence of proximal contextual factors (i.e., parental responsiveness) in primary education on motivational factors in lower-secondary education (educational effort) and indicators of lack of goal progress in upper-secondary education (premature contract terminations, failed attempts in the qualification procedure, lack of an upper-secondary diploma). Parental responsiveness’ effect in primary education on educational effort in lower-secondary education and indicators of lack of goal progress in upper-secondary education is assumed to also represent parental responsiveness’ effects at lower- and upper-secondary levels due to high levels of relative stability in parental responsiveness over time (Forehand and Jones 2002; Zhang et al. 2017). The following hypotheses were tested:

##### Hypothesis 5

The greater primary school children’s perceived parental responsiveness is, the greater is their educational effort as adolescents in lower-secondary education (H5a), the lower is their number of premature contract terminations (H5b), and the less probable is their risk of having one or more failed attempt(s) in the qualification procedure (H5c).

Primary school children’s parental responsiveness has a negative indirect effect on the probability of lacking an upper-secondary diploma 5 years after completion of compulsory school, simply mediated by the number of premature contract terminations (H5d), simply mediated by having one or more failed attempt(s) in the qualification procedure (H5e), serially mediated by educational effort in lower-secondary education and the number of premature contract terminations (H5f), and serially mediated by educational effort in lower-secondary education and having one or more failed attempt(s) in the qualification procedure (H5g).

### Control variables

#### Delayed entry into VET

**Fig. 1:**
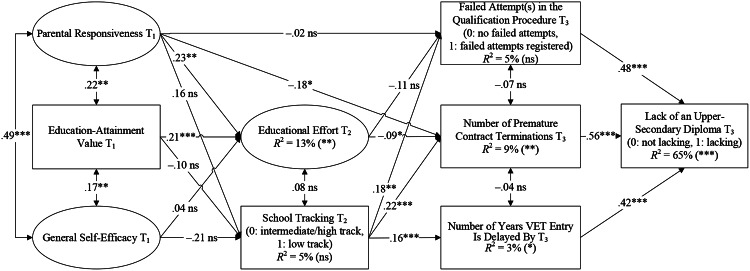
SEM to predict the lack of a diploma at the upper-secondary level. Note Standardized coefficients; measurement models and one correlation (failed attempts in the qualification procedure T_3_ with number of years VET entry is delayed by T_3_, –0.20*) are not illustrated for better readability. *N* = 1,779; T_1_ = Timepoint 1 (autumn 2011, 5th grade, primary education), T_2_ = Timepoint 2 (spring 2016, 9th grade, lower-secondary education), T_3_ = Timepoint 3 (between August 2016 and December 2021, VET, upper-secondary education), *R*^2^ = explained variance. *p < .05 (one-tailed), **p < .01 (one-tailed), ***p < .001 (one-tailed), ns = nonsignificant

A delayed VET entry occurs when adolescents enter upper-secondary education not directly after completing compulsory school (Schmid 2017). In Switzerland, about 5% of a cohort have a delayed VET entry (SFSO, 2016). The later adolescents enter their initial VET program after lower-secondary education, the more likely they are to lack an upper-secondary diploma some years after completing compulsory school (e.g., 6 years after completing compulsory school; Stalder et al. 2008). There are several possible reasons for a delayed transition, such as attending the 10th school year, choosing an interim solution, being in a preapprenticeship, and working without any upper-secondary diploma. The vast majority of individuals in one of these transitional programs lack goal progress because they are not in a qualifying upper-secondary education program. However, unlike premature contract terminations and failed attempts in the qualification procedure, this lack of goal progress does not occur at the upper-secondary level but before. Therefore, it also does not represent a nonlinear career trajectory at upper-secondary level. Besides, most individuals in these transitional programs remain in the education system, which helps them stay on track to achieve an upper-secondary diploma. After premature contract terminations or failed attempts in the qualification procedure, individuals fall out of the education system, at least temporarily. In consequence, we did not formulate hypotheses regarding delayed VET entry but included it as a control variable in our research model (see Fig. 1). We controlled the effect of the number of years VET entry is delayed by on the lack of an upper-secondary diploma 5 years after completion of compulsory school.

#### School tracking

School tracking, in some studies also referred to as compulsory school leaving certificate (e.g., Holtmann and Solga 2023), plays a vital role in explaining the lack of goal progress in upper-secondary education. Research has shown that adolescents in low tracks face more adverse conditions in achieving their career goals. Among other things, they have to choose their training occupation from a limited number of options due to their status as low-track students (Holtmann and Solga 2023). Consequently, problems concerning the fit between VET environment and personal preferences are likely to occur. Additionally, being in a low school track is associated with more learning problems in VET due to lower levels of cognitive and/or socioemotional skills (Holtmann and Solga 2023). Recent studies have shown that the risk of premature contract termination is higher for adolescents who were in a lower track in lower-secondary education than for adolescents who were in intermediate or higher tracks (Böhn and Deutscher 2022; Michaelis and Richter 2022). Furthermore, students in lower-secondary school tracks show a higher probability of a delayed transition to upper-secondary education (Düggeli 2017). In SCCT, school tracking is a distal contextual factor and therefore not part of our research questions. We did not formulate hypotheses for school tracking but used it as a control variable in our research model. In accordance with the previous research, we specified paths from school tracking to number of premature contract terminations, failed attempt(s) in the qualification procedure, and the number of years VET entry is delayed by.

Regarding general self-efficacy, education-attainment value, and parental responsiveness, we specified paths from all of these concepts to school tracking. We based this decision on research that supports their effects on academic achievements and grades. Achievements and grades strongly predict school track assignment (e.g., Neuenschwander 2013). A meta-analysis by Valentine et al. (2004) has shown that self-beliefs (e.g., general self-efficacy expectations) enhance academic achievement. Recent studies have provided evidence for positive correlations between education-attainment value and academic achievements among primary school children (Li et al. 2021; Metsäpelto et al. 2017; Putwain et al. 2019). In a meta-analysis, Pinquart (2016) identified a positive correlation between parental responsiveness and children’s academic achievements.

## Methods

### Data sources

We tested our research model using data from our multiwave research project “Effects of Tracking” (Neuenschwander et al. 2024a), complemented with data from the multiwave research project “Longitudinal Analyses in the Education Sector” (SFSO 2024). In both research projects, data contained information on the same individuals.

#### Effects of tracking

In our multiwave research project, we asked adolescents from four Swiss cantons to fill out questionnaires at multiple points in compulsory school and upper-secondary education to investigate transitions in the Swiss educational system. The research project started in autumn 2011, when the participants were in fifth grade (Wave 1). Three waves followed in compulsory school, with Wave 2 in sixth grade (autumn 2012), Wave 3 in seventh grade (autumn 2013), and Wave 4 in ninth grade (spring 2016). Because Wave 1 was the only wave in the research project that contained information about all participants’ experiences in primary education and Wave 4 covered the situation at the end of lower-secondary education, Wave 1 and Wave 4 data were used in this study. In Wave 1 (Time 1 [T_1_]), 1,735 adolescents participated. Wave 4 (Time 2 [T_2_]) consisted of 2,376 participants. In total, 474 adolescents participated at T_1_ and T_2_, 1,261 adolescents only participated at T_1_, and 1,902 adolescents only participated at T_2_.

As part of the sampling process to obtain the original study sample, schools in the Swiss Cantons of Aargau, Basel-Land, Berne, and Lucerne with fifth grade classes were asked to participate in the study. In total, 1,802 adolescents were approached, of whom 1,735 participated in the survey. In the following waves, if adolescents changed classes, all students in those classes were asked to participate in the study. In T_2_, additional ninth grade classes in the study cantons that had not already participated in prior waves were asked to participate in the study to increase the sample size. This supplementary sample consisted of 1,483 adolescents, who were added to the 893 adolescents who participated in prior survey waves. In T_1_ and T_2_, adolescents filled out paper questionnaires in their classrooms during school hours. In T_2_, the supplementary sample filled out an online questionnaire at home. For participants who filled out the questionnaire in their classrooms, members of the research team (T_1_) and teachers (T_2_) supervised the survey after receiving a detailed manual. The supplementary sample received a personalized password to access the online survey. Participants received a small incentive after study participation at T_1_. The T_2_ survey did not contain incentives. The study was conducted according to and in line with the guidelines of the institutional review board. Questionnaires met ethical standards. We obtained informed consent from the adolescents’ parents at T_1_ and T_2_. At both measurement points, the surveyed people explicitly and voluntarily agreed to participate. Refusal to participate in the study was not tied to any negative consequences. Prior to both surveys, we pretested the questionnaires on same-aged adolescents and revised the questionnaire based on their feedback. Those adolescents did not subsequently take part in the actual surveys, and their data was deleted. Including instructions, 90 min were allotted for completing the T_1_ survey. For the T_2_ survey, participants took an average of about 60 min to answer all questions. According to our research, at the time of publication, “Effects of Tracking” was the only school-based multiwave project in Switzerland that contained data on the primary and lower-secondary levels from various cantons and allowed for matching datasets with other project data.

#### Longitudinal analyses in the education sector

“Longitudinal Analyses in the Education Sector” was launched with the aim of reconstructing and contextualizing the pathways in the Swiss education system (SFSO 2024). It contains data from the “Statistics on Vocational Education and Training,” which were used in this study. The statistics include all people who are enrolled in VET and provide the year of training they are enrolled in, their number of premature contract terminations, and their results in the qualification procedure. The statistics come from an annual full census. The SFSO receives the data in electronic form from the cantonal VET offices, which are obliged to provide information by law. Data from this project were used because the project employs the only database in Switzerland that contains full information on adolescents’ educational pathways.

#### Data matching

Data from both research projects, “Effects of Tracking” and “Longitudinal Analyses in the Education Sector,” were matched to one dataset using a pseudonymizing identifier variable. The matching was based on all 2,376 Wave 4 participants in “Effects of Tracking.” Data matching was possible for 96% of adolescents (*n* = 2,279) who represent 25% (*n* = 442) of all Wave 1 participants. Data from “Longitudinal Analyses in the Education Sector” provided information about adolescents’ educational attainment situation for the period between August 2016 and December 2021 (Time 3 [T_3_]).

### Sample

To answer our research questions, we selected those adolescents who were in a VET program (dual VET or full-time vocational school) at some point between August 2016 and December 2021 (*N* = 1,803). The sample choice was based on data from “Longitudinal Analyses in the Education Sector” because the project contains full census data. If adolescents earned an upper-secondary diploma in an academic/general upper-secondary school before starting their training in VET (*n* = 14), they were excluded from the study sample because striving to achieve a diploma in academic/general and vocational upper-secondary education is uncommon and could cause model estimation biases. We also excluded adolescents who obtained an academic/general upper-secondary diploma after leaving VET without a diploma (*n* = 6) because those adolescents meet the 95% upper-secondary completion rate objective and therefore are not part of our research focus. Last, we excluded adolescents when they met all of the following criteria because meeting all criteria indicates a scenario that is not possible and thus points to inconsistencies in data entry (*n* = 4): (a) without any upper-secondary diploma, (b) no premature contract terminations, (c) no failed attempts in the qualification procedure, (d) start of VET program of 3 or 4 years in 2017 at the latest or start of VET program of 2 years in 2019 at the latest. The final sample size consisted of 1,779 adolescents (Table 1).

**Table 1:** Final study sample

Sample	*n* (total)	*n* (female)	*n* (male)	*n* (NGI)	Age at T_2_ (*M*)	Age at T_2_ (*SD*)
Total	1,779	889	890	0	15.7 years	0.6 years
T_1_ + T_2_	323	156	167	0	15.6 years	0.5 years
T_1_ only	0	0	0	0	—	—
T_2_ only	1,456	729	724	3	15.8 years	0.6 years

Note. NGI = No gender indicated at the time of measurement; T_1_ = Timepoint 1 (Wave 1, autumn 2011, 5th grade, primary education), T_2_ = Timepoint 2 (Wave 4, spring 2016, 9th grade, lower-secondary education). For all 1,779 adolescents, T_3_ data were available (except for three cases with missing data in variable number of years VET entry is delayed by)

We conducted Little’s test of missing completely at random in SPSS (Version 28). The test yielded insignificant results, χ^2^ (502, *N* = 1,779) = 522.35, *p* = .256, indicating that missing values were completely at random. In addition, we tested for missing response patterns between those who participated in T_1_ and T_2_ and those who only participated in T_2_ by conducting *t* tests and χ^2^ tests with SPSS (Version 28) for all T_2_ and T_3_ items that we used in the study. Significant but small differences arose in educational effort, Item 1: *t*(1,753) = 2.24, *p* = .025, Cohens *d* = 0.137, 95% confidence interval (CI) [0.017, 0.262]; Item 2: *t*(1,752) = 2.67, *p* = .008, Cohens *d* = 0.167, 95% CI [0.044, 0.289]; Item 3: *t*(1,748) = 2.70, *p* = .007, Cohens *d* = 0.168, 95% CI [0.046, 0.290]; Item 4: *t*(1,748) = 3.30, *p* < .001, Cohens *d* = 0.206, 95% CI [0.083, 0.328]; school tracking, χ^2^ (1, *N* = 1,718) = 17.05, *p* < .001, φ = 0.100; and number of premature contract terminations, *t*(558.62) = − 1.98, *p* = .048, Cohens *d* = − 0.107, 95% CI [–0.228, 0.014]. No response biases emerged for number of years VET entry is delayed by, *t*(1,774) = 0.01, *p* = .996, Cohens *d* = 0.000, 95% CI [–0.120, 0.121]; failed attempt(s) in the qualification procedure, χ^2^ (1, *N* = 1,779) = 2.63, *p* = .105, φ = 0.038; or lack of an upper-secondary diploma, χ^2^ (1, *N* = 1,779) = 2.88, *p* = .090, φ = 0.040. According to Graham (2009), structural equation models with a missing data feature (as applied in this study) may be used in conjunction with longitudinal data as long as missing-data procedures preserve variances, covariances, and means (i.e., estimates without bias). The insignificant results from Little’s test of missing completely at random and the small effect sizes from the missing response patterns’ test indicate that the estimation of missing values in conjunction with our longitudinal data produced reliable outcomes (Graham 2009).

### Instruments

We assessed adolescents’ general self-efficacy, education-attainment value, parental responsiveness, educational effort, and school tracking in “Effects of Tracking.” Information on adolescents’ delayed entry into VET, premature contract terminations, failed attempt(s) in the qualification procedure, and lack of an upper-secondary diploma were taken from “Longitudinal Analyses in the Education Sector.”

#### General self-efficacy

We surveyed adolescents’ general self-efficacy in primary education (fifth grade; T_1_). Adolescents completed a shortened version of the General Self-Efficacy Scale (Schwarzer and Jerusalem 1995) and rated six items on a 6-point Likert scale from 1 (*not true at all*) to 6 (*completely true*). A sample item is “It is easy for me to stick to my aims and accomplish my goals.” Between 312 and 316 adolescents responded to the six items (missing values: *n* = 1,463–1,467, 82%; factor: *N* = 320, *M* = 4.27, *SD* = 0.70, α = 0.77, range: 1.50–5.67). Schwarzer and Jerusalem (1995) assessed the General Self-Efficacy Scale with data from Germany (students and mostly adults) and reported Cronbach’s alpha values between 0.82 and 0.93.

#### Education-attainment value

Adolescents rated the value they ascribed to education for their current learning context of primary school (fifth grade, T_1_). We developed one item based on Ford (1995): “How important is school to you?” Adolescents were asked to complete the sentence “School is…” and respond on a scale from 1 (*not important at all*) to 6 (*extremely important*). In total, 303 adolescents responded (missing values: *n* = 1,476, 83%; *M* = 5.34, *SD* = 0.70, range: 3–6). We included this variable in our research model and labeled it “Education-attainment value.”

#### Parental responsiveness

Parental responsiveness was assessed when adolescents were in primary education (fifth grade, T_1_) using a shortened version of the Authoritative Parenting Index (Jackson et al. 1998). Adolescents responded to six items measuring the responsiveness dimension (e.g., “My parents make me feel better when I am upset.”) on a 6-point Likert scale from 1 (*not true at all*) to 6 (*completely true*). Between 315 and 320 adolescents completed items (missing values: *n* = 1,459–1,464, 82%; factor: *N* = 320, *M* = 5.33, *SD* = 0.59, α = 0.85, range: 2–6). Jackson et al. (1998) reported a Cronbach’s alpha value of 0.85 for the responsiveness subscale in two U.S. studies with elementary and middle school children (Study 1) and high school students (Study 2).

#### Educational Effort

We assessed adolescents’ educational effort in lower-secondary education (ninth grade, T_2_). Adolescents responded to four items on a 6-point Likert scale from 1 (*not true at all*) to 6 (*completely true*). The items were adopted from Neuenschwander et al. (2013) and represent a modified version by Schmidt et al. (1998). A sample item is “I am really hardworking at school.” Between 1,750 and 1,755 adolescents provided information on items (missing values: *n* = 24–29, 1–2%; factor: *N* = 1,755, *M* = 4.20, *SD* = 0.94, α = 0.89, range: 1–6).

#### School tracking

Adolescents reported their school level in lower-secondary education with one item (ninth grade, T_2_). They were asked to answer the question “What school level are you attending now?” with response options differing depending on the canton. In total, 1,718 adolescents provided information on their school level (missing values: *n* = 61, 3%). Responses were grouped into three categories: 1 (low track, *n =* 506), 2 (intermediate track, *n =* 910), and 3 (high track, *n =* 302). For this study, we dummy coded responses into a new variable as 0 (intermediate or high track, *n* = 1,212 [71% of valid values]) or 1 (low track, *n* = 506 [29% of valid values]).

#### Number of years VET entry is delayed by

Adolescents’ delayed entry into VET was included in the analyses with one item. We counted the number of years that passed between a cohort’s regular completion of compulsory school in the summer of 2016 and individuals’ initial VET entry within the period between August 2016 and December 2021 (T_3_). Direct entry into VET in autumn 2016 was coded as 0 years, entry in 2017 as 1 year, entry in 2018 as 2 years, and so on. The continuous variable contained information for 1,776 adolescents (missing values: *n* = 3, < 1%; *M* = 0.36, *SD* = 0.68, range: 0–4).

#### Number of premature contract terminations

The number of premature contract terminations was included in the analyses with one item. We counted the number of premature contract terminations between August 2016 and December 2021 (T_3_) but only for the time it took individuals to acquire their first upper-secondary diploma. The continuous variable contained information for all 1,779 adolescents (missing values: *n* = 0, 0%; *M* = 0.20, *SD* = 0.49, range: 0–3).

#### Failed attempt(s) in the qualification procedure

We included adolescents’ failed attempt(s) in the qualification procedure with one item. We counted the number of failed attempts between August 2016 and December 2021 (T_3_) but only for the time it took individuals to acquire their first upper-secondary diploma. Information was available for all 1,779 adolescents (missing value: *n* = 0, 0%). In total, 1,713 adolescents did not have any failed attempts registered, 59 adolescents had one failed attempt, six adolescents had two failed attempts, and one adolescent had three failed attempts. In this study, we dummy coded a new categorical variable as 0 (no failed attempts registered, *n* = 1,713 [96%]) or 1 (failed attempt[s] registered, *n* = 66 [4%]).

#### Lack of an upper-secondary diploma

The lack of an upper-secondary diploma was included in the analyses with one item. We checked whether adolescents completed their VET program with a diploma/certificate between August 2016 and December 2021 (T_3_). We had information for all 1,779 adolescents (missing values: *n* = 0, 0%). In a new categorical variable, we assigned those adolescents with a diploma the value 0 (not lacking, *n* = 1,620 [91%]) and those adolescents without a diploma the value 1 (lacking, *n* = 159 [9%]). We only coded adolescents as 0 if they acquired their diploma in the Swiss educational system.

### Analytical procedure

We examined the research model using structural equation modeling (SEM) in Mplus (Version 8.1). SEM allows for latent variable estimation which builds on the use of multiple indicators that contain measurement errors. Latent variables are thus corrected for measurement errors which makes SEM an analytical method with a higher reliability compared to other methods. SEM enables researchers to analyze complex variable relationships (Skrondal and Rabe-Hesketh 2004). Using a simultaneous confirmatory factor analysis (SCFA), we tested measurement models for configural invariance. Configural invariance indicates a fit between the theoretical model and data; it is required for SEM. We determined configural invariance using various model fit indices: the comparative fit index (CFI), root mean square error of approximation (RMSEA), and standardized root mean square residual (SRMR). CFI ≥ 0.95 indicates an acceptable fit. RMSEA and SRMR ≤ 0.08 and 0.10, respectively, are considered indications of an acceptable fit (Schermelleh-Engel et al. 2003). When the indices’ values did not meet the criteria, we modified the model parsimoniously step by step on the basis of modification indices and item wording. As a preliminary analysis, we then calculated bivariate correlations for each pair of variables in separate models in Mplus. General self-efficacy, parental responsiveness, and educational effort were treated as latent variables with the measurement models from the SCFA. In SEM, we applied measurement models from the invariance tests, and we specified paths in accordance with the proposed research model and considering the effects of and on control variables (school tracking, number of years VET entry is delayed by). We specified correlations between same-wave variables (Bollen and Noble 2011). Because there are several reasons for a delayed VET entry, of which some are independent of adolescents’ effort, the path from educational effort to number of years VET entry is delayed by was constrained at zero. Mediation hypotheses were tested under the mediation-only assumption. Consequential, direct paths from T_1_ variables to number of years VET entry was delayed by, number of premature contract terminations, and failed attempt(s) in the qualification procedure were constrained at zero except for the direct paths postulated in the hypotheses from parental responsiveness to number of premature contract terminations and failed attempt(s) in the qualification procedure. Additionally, direct paths from T_1_ and T_2_ variables to lack of an upper-secondary diploma were constrained at zero. We used the weighted least square mean and variance adjusted estimation with theta parametrization. The pairwise present method in Mplus, which is used as a standard with this form of estimation, was applied to deal with missing values. We used the same index criteria to determine model fit for SEM as we did for the configural invariance test. When the indices’ values did not meet the criteria, we parsimoniously modified the model step by step and changed structural model parameters on the basis of modification indices. Indirect effects and effect size differences were tested in Mplus with the “model indirect” feature and with a Wald test, respectively. For the mediation analysis, we used the bias-corrected bootstrapping method to compute confidence intervals more accurately (MacKinnon 2008). We reported all correlation and regression coefficients with one-tailed *p* values.

## Results

### Test of configural invariance

We tested the measurement models for configural invariance using SCFA. The baseline model fit the data, χ^2^(101, *N* = 1,766) = 173.99, *p* < .001, CFI = 0.98, RMSEA = 0.02, SRMR = 0.05, which indicates configural invariance. However, because the standardized factor loading of one item measuring general self-efficacy (Item 1: “If something opposes me, I can find the means and ways to get what I want.”) fell below the threshold value of 0.4 in the final SEM (Brown 2006), we recalculated the measurement model without that item, resulting in a slightly better model fit, χ^2^(87, *N* = 1,766) = 151.85, *p* < .001, CFI = 0.99, RMSEA = 0.02, SRMR = 0.05. In all subsequent analyses, we applied the measurement model without that item.

### Bivariate correlations

Table 2 reports bivariate correlations for study variables. The lack of an upper-secondary diploma had a statistically significant correlation with all study variables except for parental responsiveness, *r*(1,777) = –0.02, *p* = .415, and general self-efficacy, *r*(1,777) = –0.07, *p* = .185. Positive correlations occurred with failed attempt(s) in the qualification procedure, *r*(1,777) = 0.13, *p* < .001; the number of premature contract terminations, *r*(1,777) = 0.54, *p* < .001; the number of years VET entry was delayed by, *r*(1,777) = 0.30, *p* < .001; and school tracking, *r*(1,777) = 0.10, *p* < .001. Negative correlations occurred with educational effort, *r*(1,777) = –0.10, *p* < .001, and education-attainment value, *r*(1,777) = –0.15, *p* = .013.

**Table 2:** Correlations for study variables

Variable	1	2	3	4	5	6	7	8
1. Lack of an upper-secondary diploma T_3_^a^	—							
2. Failed attempt(s) in the qualification procedure T_3_^b^	0.13***	—						
3. Number of premature contract terminations T_3_	0.54***	0.01	—					
4. Number of years VET entry is delayed by T_3_	0.30***	–0.05*	–0.00	—				
5. School tracking T_2_^c^	0.10***	0.07**	0.17***	0.14***	—			
6. Educational effort T_2_	–0.10***	–0.01	–0.11***	–0.03	0.05*	—		
7. Parental responsiveness T_1_	–0.02	–0.04	–0.18**	0.03	0.01	0.28***	—	
8. Education-attainment value T_1_	–0.15*	–0.09	–0.21***	0.08	–0.02	0.26***	0.20***	—
9. General self-efficacy T_1_	–0.07	–0.02	–0.18**	–0.11*	–0.10	0.17**	0.44***	0.16**

Note. ^a^ 0 = not lacking (i.e., diploma acquired) and 1 = lacking (i.e., no diploma acquired [yet]), ^b^ 0 = no failed attempt(s) registered and 1 = failed attempt(s) registered, ^c^ 0 = intermediate or high track and 1 = low track. T_1_ = Timepoint 1 (autumn 2011, 5th grade, primary education), T_2_ = Timepoint 2 (spring 2016, 9th grade, lower-secondary education), T_3_ = Timepoint 3 (between August 2016 and December 2021, VET, upper-secondary education). *N* = 320–1,779. Variables are metrically scaled unless otherwise stated

*p < .05 (one-tailed), **p < .01 (one-tailed), ***p < .001 (one-tailed)

### Testing the research model with SEM

We fit the structural equation model to the data, χ^2^(172, *N* = 1,779) = 254.84, *p* < .001, CFI = 0.97, RMSEA = 0.02, SRMR = 0.05. Figure 1 shows the model results (standardized coefficients β).

Regarding our first research question, the adolescents’ number of premature contract terminations and failed attempt(s) in the qualification procedure had a statistically significant positive direct effect on lack of an upper-secondary diploma (β = 0.56, 95% CI [0.48, 0.65], *p* < .001 and β = 0.48, 95% CI [0.26, 0.65], *p* < .001, respectively; Hypotheses 1a and 1b are supported). The Wald test showed that the effect size of adolescents’ number of premature contract terminations on lack of an upper-secondary diploma is significantly bigger than the effect size of failed attempt(s) in the qualification procedure on lack of an upper-secondary diploma, ∆χ^2^(1, *N* = 1,779) = 65.91, *p* < .001 (Hypothesis 1c is supported). Regarding our second research question, adolescents’ educational effort in lower-secondary education had a statistically significant negative direct effect on number of premature contract terminations (β = − 0.09, 95% CI [–0.17, 0.00], *p* = .017; Hypothesis 2a is supported) but had an insignificant negative direct effect on failed attempt(s) in the qualification procedure (β = − 0.11, 95% CI [–0.30, 0.10], *p* = .125; Hypothesis 2b is rejected). Accordingly, adolescents’ educational effort had a statistically significant negative indirect effect on lack of an upper-secondary diploma, simply mediated by number of premature contract terminations (β_Ind_ = − 0.05, 95% CI [–0.11, − 0.00], *p* = .023; Hypothesis 2c is supported) but not simply mediated by failed attempt(s) in the qualification procedure (β_Ind_ = − 0.06, 95% CI [–0.16, 0.03], *p* = .135; Hypothesis 2d is rejected). Regarding primary education predictors, adolescents’ general self-efficacy did not have a statistically significant positive direct effect on their educational effort in lower-secondary education (β = 0.04, 95% CI [–0.13, 0.19], *p* = .319; Hypothesis 3a is rejected). Accordingly, adolescents’ general self-efficacy had insignificant negative indirect effects on lack of an upper-secondary diploma, serially mediated by educational effort and number of premature contract terminations (β_Ind_ = − 0.00, 95% CI [–0.02, 0.01], *p* = .338; Hypothesis 3b is rejected) and serially mediated by educational effort and failed attempt(s) in the qualification procedure (β_Ind_ = − 0.00, 95% CI [–0.03, 0.01], *p* = .379; Hypothesis 3c is rejected). In contrast, adolescents’ education-attainment value in primary education had a statistically significant positive direct effect on their educational effort (β = 0.21, 95% CI [0.09, 0.33], *p* < .001; Hypothesis 4a is supported). Their education-attainment value had a statistically significant negative indirect effect on lack of an upper-secondary diploma, serially mediated by educational effort and number of premature contract terminations (β_Ind_ = − 0.01, 95% CI [–0.03, − 0.00], *p* = .047; Hypothesis 4b is supported) but not serially mediated by educational effort and failed attempt(s) in the qualification procedure (β_Ind_ = − 0.01, 95% CI [–0.04, 0.01], *p* = .150; Hypothesis 4c is rejected). Primary school children’s parental responsiveness had a statistically significant positive direct effect on educational effort (β = 0.23, 95% CI [0.08, 0.39], *p* = .002; Hypothesis 5a is supported). Further, parental responsiveness had a statistically significant negative direct effect on number of premature contract terminations (β = − 0.18, 95% CI [–0.36, 0.03], *p* = .035; Hypothesis 5b is supported) but insignificantly predicted failed attempt(s) in the qualification procedure (β = − 0.02, 95% CI [–0.52, 0.58], *p* = .471; Hypothesis 5c is rejected). Parental responsiveness had a statistically significant negative indirect effect on lack of an upper-secondary diploma, simply mediated by number of premature contract terminations (β_Ind_ = − 0.10, 95% CI [–0.20, 0.02], *p* = .034; Hypothesis 5d is supported) but not simply mediated by failed attempt(s) in the qualification procedure (β_Ind_ = − 0.01 95%, CI [–0.22, 0.25], *p* = .467; Hypothesis 5e is rejected). Furthermore, parental responsiveness had a statistically significant negative indirect effect on lack of an upper-secondary diploma, serially mediated by educational effort and number of premature contract terminations (β_Ind_ = − 0.01, 95% CI [–0.03, − 0.00], *p* = .042; Hypothesis 5f is supported) but not serially mediated by educational effort and failed attempt(s) in the qualification procedure (β_Ind_ = − 0.01, 95% CI [–0.05, 0.00], *p* = .186; Hypothesis 5g is rejected). Table 3 summarizes all hypothesized indirect effects on lack of an upper-secondary diploma. Overall, the explained variance in lack of an upper-secondary diploma was 65%.

**Table 3:** Hypothesized indirect effects on lack of an upper-secondary diploma

Indirect paths to Lack of an upper-secondary diploma		95% CI		
	β_Ind_	Lower	Upper	*p*
Effort → Premature contract termination	–0.05	–0.11	–0.00	0.023
Effort → Failed attempt(s) in the QP	–0.06	–0.16	0.03	0.135
Self-efficacy → Effort → Premature contract termination	–0.00	–0.02	0.01	0.338
Self-efficacy → Effort → Failed attempt(s) in the QP	–0.00	–0.03	0.01	0.379
Value → Effort → Premature contract termination	–0.01	–0.03	–0.00	0.047
Value → Effort → Failed attempt(s) in the QP	–0.01	–0.04	0.01	0.150
Responsiveness → Premature contract termination	–0.10	–0.20	0.02	0.034
Responsiveness → Failed attempt(s) in the QP	–0.01	–0.22	0.25	0.467
Responsiveness → Effort → Premature contract termination	–0.01	–0.03	–0.00	0.042
Responsiveness → Effort → Failed attempt(s) in the QP	–0.01	–0.05	0.00	0.186

Note. *N* = 1,779. QP = Qualification procedure. One-tailed *p*-values

## Discussion

### Summary and interpretation

In the present study, we investigated predictors of adolescents’ situation of lacking an upper-secondary diploma 5 years after they complete compulsory school. We analyzed the predictive share of two of the main events indicating nonlinear career trajectories at upper-secondary level—premature contract terminations and failed attempts in the qualification procedure. Building on SCCT, we also examined the extent to which adolescents’ general self-efficacy, education-attainment value (both motivational factors), and parental responsiveness (a proximal contextual factor) in primary education as well as their educational effort in lower-secondary education (another motivational factor) predict the lack of a vocational upper-secondary diploma, controlling for the effects of school tracking in lower-secondary education and delays in VET entry. To our knowledge, for the first time, adolescents’ probability of lacking an upper-secondary diploma was investigated with predictors that were measured as early as primary education (i.e., 10 years before the measurement of the dependent variable).

With SEM applied, the results showed that premature contract terminations and failed attempts in the qualification procedure predict adolescents’ probability of lacking an upper-secondary diploma. The effect size of premature contract terminations was bigger than that of failed attempts in the qualification procedure. Additionally, adolescents’ educational effort in lower-secondary education predicted their number of premature contract terminations in upper-secondary education, which mediated effort’s effect on their probability of lacking an upper-secondary diploma. This finding aligns with previous research (Findeisen et al. 2023; Vasalampi et al. 2010).

Of the three primary school predictors, adolescents’ education-attainment value and their parents’ responsiveness predicted the lack of an upper-secondary diploma, serially mediated by educational effort in lower-secondary education and premature contract terminations in upper-secondary education. Results also pointed to a tendency that the responsiveness of adolescents’ perceived authoritative parenting style could have exerted a direct effect on number of premature contract terminations, influencing the probability of lacking an upper-secondary diploma, simply mediated by premature contract terminations as well. In view of the confidence intervals, this result should be interpreted with caution. Previous research supports these effects of parental responsiveness (e.g., Blondal and Adalbjarnardottir 2009, 2014; Tuominen et al. 2020).

Contrary to our hypotheses, adolescents’ probability of having one or more failed attempt(s) in the qualification procedure could not be predicted by their educational effort in lower-secondary school or by their parents’ responsiveness in primary education. Consequently, adolescents’ educational effort in lower-secondary education did not indirectly affect the probability of lacking an upper-secondary diploma 5 years after they completed compulsory school, simply mediated by having one or more failed attempt(s) in the qualification procedure, and parental responsiveness and their education-attainment value in primary school did not indirectly predict lacking an upper-secondary diploma, neither serially mediated by educational effort in lower-secondary education and having one or more failed attempt(s) in the qualification procedure nor simply mediated by having one or more failed attempt(s) in the qualification procedure (for parental responsiveness). Apparently, adolescents’ probability of having one or more failed attempt(s) in the qualification procedure cannot be predicted by factors that are measured prior to adolescents’ VET entry. Instead, those failed attempts seem to stem more from experiences adolescents gain in their upper-secondary education program. Yet, to the best of our knowledge, there is no research on determinants of failed attempts in the qualification procedure that could support this statement. We therefore call for research in this area. Although the impact of number of premature contract terminations is more substantial, failing the qualification procedure accounts for a significant proportion of the lack of an upper-secondary diploma.

Furthermore, adolescents’ general self-efficacy in primary school did not predict their educational effort in lower-secondary education and therefore did not exert any effect on the probability of lacking an upper-secondary diploma 5 years after completion of compulsory school. Previous research supported positive correlations between general self-efficacy and educational effort (Neuenschwander et al. 2024b; Vasalampi et al. 2010). One reason for the insignificant results could be situated in the self-efficacy measurement’s specificity. We see potential in using slightly different self-efficacy measures, such as educational self-efficacy, academic self-efficacy, and school self-efficacy (e.g., Jerusalem and Satow 1999). All of these self-efficacy measures relate more to our study sample’s educational context (primary school children on their way to becoming adolescents in lower- and upper-secondary education). Additionally, measures are closer to goal self-efficacy, as proposed in SCCT (Lent and Brown 2008). Research has shown significant correlations between these self-efficacy measures and educational effort or similar concepts. For example, in a study applying (educational) work-avoidance goals, which is a similar but inversely coded concept to educational effort, Schweder et al. (2022) found a statistically significant negative correlation with goal self-efficacy among female lower-secondary students. Steinmayr et al. (2018) found statistically significant correlations between school self-efficacy and perseverance of effort among lower-secondary students. Kreutzmann et al. (2014) reported statistically significant positive correlations between school self-efficacy and (educational) effort among primary school children. Future studies that also employ a multiwave design from primary school to VET should use educational self-efficacy or academic self-efficacy instead of general self-efficacy to predict educational effort and the probability of lacking an upper-secondary diploma.

### Implications

Our findings have shown that the proximal contextual factor of parental responsiveness in primary education predicts lacking an upper-secondary diploma 5 years after completion of compulsory school, mostly in the form of an indirect effect serially mediated by educational effort in lower-secondary education and premature contract terminations. To reduce adolescents’ probability of lacking an upper-secondary diploma, we see a promising strategy in fostering a parenting style that is highly responsive. High levels of responsiveness increase adolescents’ educational effort, ultimately contributing to a small probability of lacking an upper-secondary diploma. Primary school children’s parents can promote responsiveness in various ways (e.g., by making their children feel better when they are upset, wanting to hear about their problems, listening to what they have to say, and telling them when they do a good job on things). In line with Böhn and Deutscher’s (2022) call of encouragement to make better use of dropout prevention programs, parental training programs may be a valuable solution to promote responsiveness. Numerous parental training programs exist to foster a parenting style with high responsiveness, such as the authoritative parenting style (e.g., ABCD Parenting Young Adolescents Program [Burke et al. 2010], Strengthening Families Programs 10–14 [Spoth et al. 2009], Adolescent Transitions Program [Connell et al. 2007], Teen Triple P Parenting Program [Ralph and Sanders 2003], and Guiding Good Choices [Haggerty et al. 1999; see Kauser and Pinquart 2019]).

Another crucial factor is education-attainment value among primary school children. Our findings show that the motivational factor of education-attainment value in primary school is highly relevant for the lack of an upper-secondary diploma, serially mediated by educational effort in lower-secondary education and premature contract terminations. To foster individuals’ education-attainment value, researchers pointed out that education-attainment value may be enhanced by teachers’ educational interests (Schiefele and Schaffner 2015) and time parents spend with their children (McNair and Johnson 2009). According to Schiefele and Schaffner (2015), the higher teachers rate their educational interests, the higher is adolescents’ education-attainment value, as measured by their subject interests. McNair and Johnson (2009) concluded that the more often adolescents’ parents engage in achievement-related activities with their adolescent children, the higher adolescents rate the importance of school and therefore value (formal) education. Teachers may foster adolescents’ education-attainment value and therefore their probability of attaining an upper-secondary diploma by having high education interests. In addition to enhancing the responsiveness in their parenting style, parents can increase adolescents’ education-attainment value and hence adolescents’ probability of achieving an upper-secondary diploma by spending more time on achievement-related activities with their children.

For future research, we suggest two research strands to pursue. First, to support our practical implications with empirical evidence, we suggest extending our research model by adding teacher interests and parents’ time spent with their child as predicators of education-attainment value. Studies in this area would not only address our implication but would also enable researchers to better address Böhn and Deutscher’s (2022) recommendation to put more focus on educators’ actions to minimize adolescents’ dropouts.

Second, researchers may analyze the process that leads people to not have an upper-secondary diploma some years after completing compulsory school more thoroughly, also considering the manifold factors in VET contexts that precede failed attempts in the qualification procedure and premature contract terminations. Indicators that measure (the lack of) goal progress prior to the qualification procedure and premature contract terminations could be adolescents’ (in)ability to meet curricular requirements and the extent to which adolescents (do not) reach their daily work goals. Incorporating those indicators of goal progress or lack thereof offers intervention options at an earlier stage of adolescents’ vocational training compared to failed attempts in the qualification procedure and premature contract terminations and may help improve the rather low explained variances in failed attempt(s) in qualification procedure und number of premature contract terminations. Research in this area responds to Böhn and Deutscher’s (2022) call for more studies on daily working and learning conditions in VET and the way adolescents deal with these conditions.

### Strengths and limitations

In this study, we successfully applied the theoretical model of the SCCT (Lent and Brown 2008) to analyze the lack of an upper-secondary diploma. Utilizing data from the Swiss Statistical Office, this multiwave study involved a large sample, and we assume good internal validity. Although we conducted this study in Switzerland, we reason that our model holds for other national VET contexts, too. This assumption is based on similar findings in other countries (e.g., for Germany, Holtmann and Solga 2023). Besides these strengths, there are some limitations.

Regarding this study’s theoretical approach, we did not include expected and received work conditions in our research model. The SCCT postulates that self-efficacy expectations and environmental supports, resources, and obstacles predict one’s expected and received work conditions, which in turn affect goal progress via effort. Previous findings suggest that work conditions is a relevant predictor of effort and goal progress. For example, Neuenschwander (2005) found a highly significant negative correlation between class size (a “work” condition in the school context) and student motivation.

A second limitation concerns the measurement of study variables. We did not include longitudinally measured variables in our research model, which would improve the measures’ validity and reliability. Although “Effects of Tracking” used a multiwave design, only a selected number of concepts are longitudinally measured due to the project’s differing research aims over time. None of the study variables was measured more than once. Further, we measured education-attainment value with only one item. Multi-item operationalizations of the concept are available (e.g., School Importance Attitudes; McNair and Johnson 2009) and would increase our findings’ validity. Due to survey questionnaire length restrictions, we choose the one-item solution based on Ford (1995). Regarding our categorical variables, we had to group some values into one category. Reducing variable values results in information loss. We grouped adolescents with two or more failed attempts in the qualification procedure with adolescents who only failed the qualification procedure once due to a low number of cases in the category. For the school tracking variable, adolescents in the intermediate and high tracks were reduced to one category to make model estimation possible.

A third limitation could be identified in the chosen time interval between completion of compulsory education and the moment of lacking an upper-secondary diploma. Although there is some evidence in favor of the 5-year period (e.g., Meyer 2018), the research design largely dictated the choice of this time interval. Researchers may want to test our model and predict the lack of an upper-secondary diploma even later in individuals’ careers (e.g., 10 years after completion of compulsory school). Later time points make it less likely that the lack of an upper-secondary diploma is just a time-of-measurement-problem. Also, obtaining information about the potential causes for the lack is more urgent at later points in individuals’ careers.

## Conclusions

Not having an upper-secondary diploma 5 years after completing compulsory school is not the end of the world. Nonetheless, noncompletion status could indicate a problematic career trajectory. At a certain age, individuals without an upper-secondary diploma increasingly become a risk group for unemployment, social welfare dependency, and undersupplying the labor market with qualified individuals. It is therefore important to identify factors that predict the probability of lacking an upper-secondary diploma to develop targeted educational policy measures that help reduce noncompletion rates. Investigating the probability of lacking a vocational upper-secondary diploma 5 years after completion of compulsory school, the present study addressed a rather underexplored topic in VET-dropout research and offered an approach for early interventions in primary and lower-secondary education.

## Data Availability

Data from the research project “Effects of Tracking” that were generated and/or analyzed during the current study are available in the Swissubase repository, at https://www.swissubase.ch/en, reference numbers 11063 and 12206. Data from the research project “Longitudinal Analyses in the Education Sector” that support this study’s results are available from the Swiss Federal Statistical Office, but restrictions apply to the availability of these data, which were used under license for the current study and so are not publicly available.

## Footnotes

1 We used the term “diploma” in this study, but upper-secondary certificates are included.

## Abbreviations

CFI: Comparative fit index
CI: Confidence interval
RMSEA: Root mean square error of approximation
SCCT: Social cognitive career theory of work satisfaction and well-being
SCFA: Simultaneous confirmatory factor analysis
SEM: Structural equation modeling
SRMR: Standardized root mean square residuals
VET: Vocational education and training
